# Characterize traction–separation relation and interfacial imperfections by data-driven machine learning models

**DOI:** 10.1038/s41598-021-93852-y

**Published:** 2021-07-12

**Authors:** Sanjida Ferdousi, Qiyi Chen, Mehrzad Soltani, Jiadeng Zhu, Pengfei Cao, Wonbong Choi, Rigoberto Advincula, Yijie Jiang

**Affiliations:** 1 Department of Mechanical Engineering, University of North Texas, Denton, TX 76207 USA; 2 Center for Nanophase Materials and Sciences, Oak Ridge National Laboratory, Oak Ridge, TN 37830 USA; 3 Chemical Sciences Division, Oak Ridge National Laboratory, Oak Ridge, TN 37831 USA; 4 Department of Materials Science and Engineering, University of North Texas, Denton, TX 76207 USA; 5 Department of Chemical and Biomolecular Engineering, University of Tennessee, Knoxville, TN 37996 USA

**Keywords:** Mechanical engineering, Mechanical properties, Composites

## Abstract

Interfacial mechanical properties are important in composite materials and their applications, including vehicle structures, soft robotics, and aerospace. Determination of traction–separation (T–S) relations at interfaces in composites can lead to evaluations of structural reliability, mechanical robustness, and failures criteria. Accurate measurements on T–S relations remain challenging, since the interface interaction generally happens at microscale. With the emergence of machine learning (ML), data-driven model becomes an efficient method to predict the interfacial behaviors of composite materials and establish their mechanical models. Here, we combine ML, finite element analysis (FEA), and empirical experiments to develop data-driven models that characterize interfacial mechanical properties precisely. Specifically, eXtreme Gradient Boosting (XGBoost) multi-output regressions and classifier models are harnessed to investigate T–S relations and identify the imperfection locations at interface, respectively. The ML models are trained by macroscale force–displacement curves, which can be obtained from FEA and standard mechanical tests. The results show accurate predictions of T–S relations (*R*^2^ = 0.988) and identification of imperfection locations with 81% accuracy. Our models are experimentally validated by 3D printed double cantilever beam specimens from different materials. Furthermore, we provide a code package containing trained ML models, allowing other researchers to establish T–S relations for different material interfaces.

## Introduction

Interfacial mechanical properties quantify adhesion interactions between two surfaces at the microscale and are critical to the performance of heterogeneous engineering materials, such as multilayer structural materials and fiber reinforced composites^1–4^. Key interfacial properties, including fracture toughness and interfacial strength, can be measured via combination of numerical simulations and multiscale physical experiments, such as standard double cantilever beam (DCB) tests to *in-situ* scanning electronic microscope (SEM) fiber pull-out tests^5–9^. In addition to parameters such as total toughness and averaged interfacial strength, establishing a traction–separation (T–S) relation can be used to fully quantify interfacial adhesion, understand crack propagation and fracture^10^, and thus lead to the novel design and fabrication of high-performance composites^11–14^.

Multiple types of T–S relations have been proposed^15–17^ based on mathematical approximations (such as Dugdale and triangular forms)^15,17,18^ or physical observations (such as Lennard–Jones potential)^19,20^. Due to the nature of short adhesion range, it requires high resolution experiments and imaging, such as atomic force microscopy (AFM)^15,18,21,22^ and optical interferometric measurements^7,10^, together with numerical and theoretical analysis to fully establish T–S relations. For example, to determine an intrinsic T–S relation of a single asperity contact, both AFM experimental pull-off forces and results from FEA and Maugis-Dugdale-*n* analytical model^23^ are obtained. Then, iterative fittings are performed using varying work of adhesion and range of adhesion to match numerical and theoretical results with experimental data and finally establish the T–S relation^15,18^. To establish T–S relations in relating with the characterized crack tip, Gowrishankar et al*.* and Wu et al*.* have performed DCB tests with *in-situ* interferometric measurements (~ 20 nm resolution) of normal crack opening displacements along with J-integral analytical solution and comparison with FEA approach^7,10^.

Recently, predictive machine learning (ML) is an emerging research area and a promising tool in prediction of the mechanical properties and design of materials^24–26^. The ML methods effectively learn on experimental or simulated data, and efficiently predict complicated data patterns or trends^27^. Supervised learning algorithm is a subset of ML methods, where models are trained with both input and output in training dataset. The underlying ML algorithms establish a pattern and predict targets based on input in testing dataset^28^. In interfacial science, recent works have advanced the prediction of interface fracture patterns, crack propagations, and interfacial thermodynamic constraints through supervised ML methods^29–33^, such as neural and deep material networks. While previous studies focus on specific types of materials, failure around known defects, and algorithm developments, still lacking are data-driven models that can establish intrinsic T–S relations and capture imperfections and can be generalized for different material interfaces.

In this study, we develop data-driven models based on FEA and ML that quantify the T–S relations and identify locations of interfacial imperfections. The established eXtreme Gradient Boosting (XGBoost) ML models provide an efficient and convenient approach for characterizing interfacial properties in a wide range of material systems. Our multi-output XGBoost models outperform other ML models and predict the microscale T–S relations with high precision (coefficient of determination *R*^2^ = 0.988) through training on macroscale force–displacement (F–D) curves. Experimental evaluations on several 3D printed material systems are performed and obtain agreeable F–D curves from experiments and FEA results using ML learned T–S relations. Additionally, to locate interfacial imperfections, a classification model is developed with an accuracy of 81 ± 8.6%. Finally, we develop a package based on the XGBoost regression model, which can facilitate the users to obtain T–S relations for their own F–D data without additional database establishment or model training.

## Results

**Figure 1:**
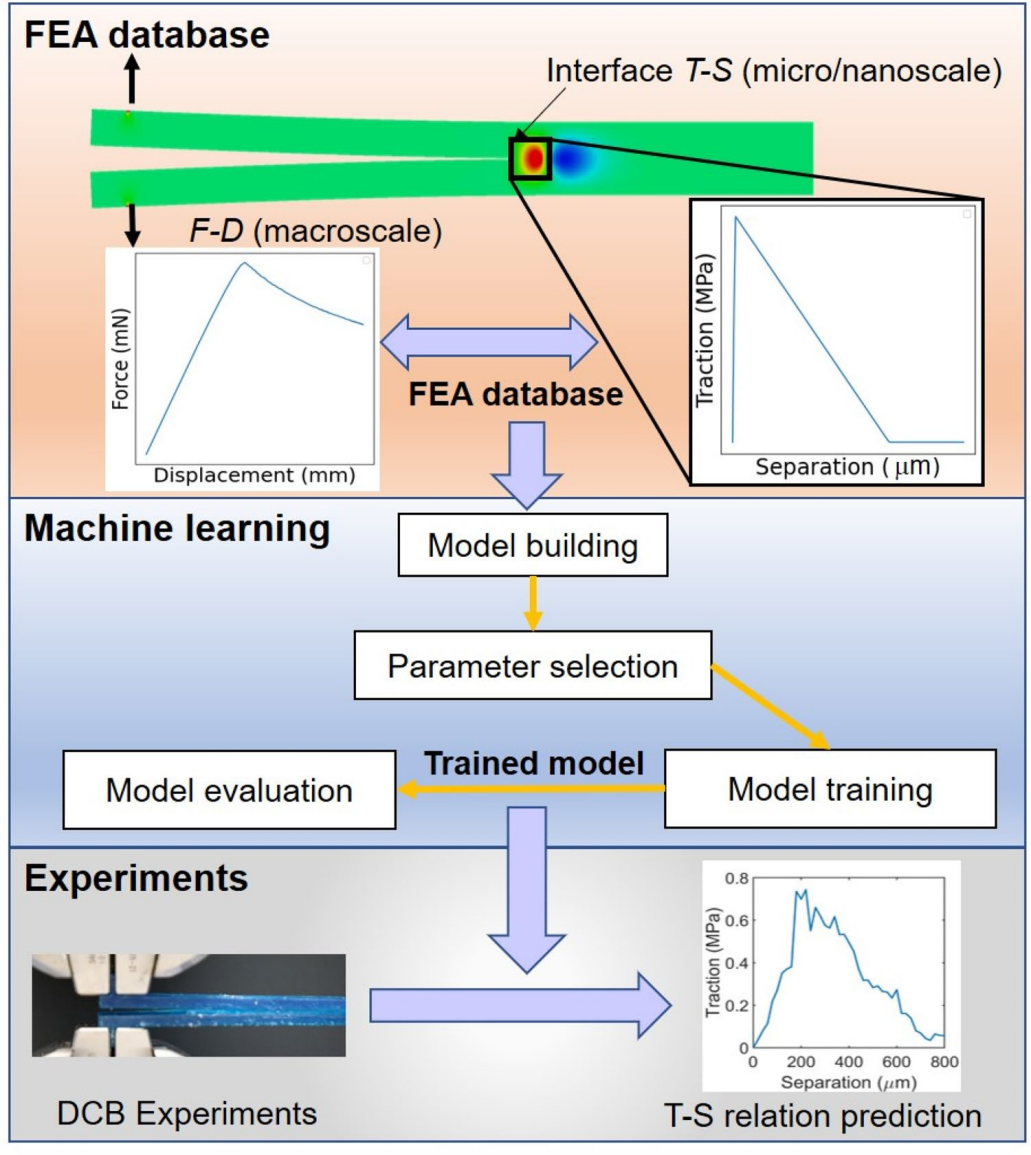
An overview schematic of the development of a data-driven method for establishing interfacial properties. The method includes FEA database generation, multi-output ML models training, and experimental validations.

An overview of our workflow is shown in Fig. 1, including three key components: (1) establishing F–D and T–S relations database by FEA; (2) training ML models; and (3) validating ML models by experimental data. Firstly, we perform FEA simulating the DCB tests to collect a series of F–D curves correlating to T–S relations with interfacial stiffness and strength spanning 5 and 6 orders of magnitude, respectively (top section of Fig. 1). Secondly, we use several ML methods (e.g., XGBoost) to train the models based on FEA database. Scalable relations from F–D curves to T–S relations are established in trained ML models (middle section of Fig. 1). Finally, we perform experimental validations (bottom section in Fig. 1) that harness the trained ML models on experimental F–D curves to establish T–S relations at the interfaces of multiple material systems.

### ML results on T–S relations

**Figure 2:**
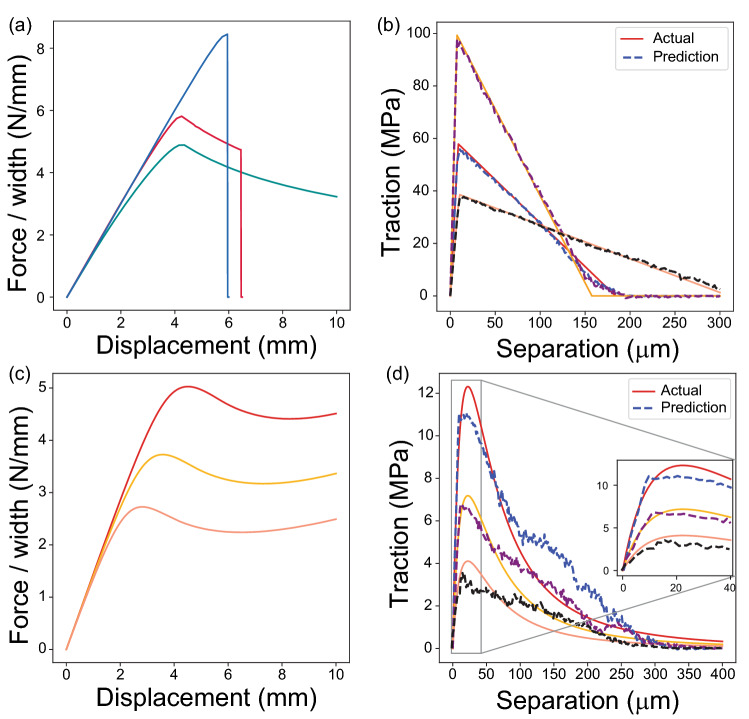
Machine learning on FEA data. (**a**) Different types of F–D curves obtained from triangular T–S relations, (**b**) comparison between triangular T–S relations predicted by XGBoost ML and the actual cases preset in FEA, (**c**) the F–D curves calculated based on different Leonard-Jones T–S relations, and (**d**) the prediction results using the F–D curves in (**c**) and comparison with preset T–S relations.

Different interfacial failure behaviors are observed from F–D curves. Several representative F–D curves are shown in Fig. 2a, including gradual crack propagation, propagation followed by catastrophic failure, and immediate failure after ultimate strength. It is challenging to correlate F–D curves and T–S relations directly as the measurements of F–D curves (macroscale) and T–S relations (microscale) are at different length scales^29^ and there is a curve to curve relation between them. However, through the training of the multi-output XGBoost model, we are able to accurately predict the interfacial T–S relations. XGBoost is a scalable version of gradient boosting framework^34^ that builds a sequential ensemble method to reduce the error of predecessor trees by updating the residual errors^35,36^. With the integration of decision tree method, XGBoost can enhance the accuracy, speed, and performance of a model. For testing dataset, the median of *R*^2^ score is 0.988, manifesting the near perfect matching case where *R*^2^ = 1. Comparisons of example T–S relations between FEA preset values and the predicted results by the XGBoost model are exhibited in Fig. 2b. The solid lines indicate the FEA preset values, whereas dashed lines represent predicted T–S relations using XGBoost model. The ML model results in accurate prediction with a triangular form and key parameters, including interfacial stiffness, strength, and range of adhesion. It is worth noting that the predicted T–S relations are output as discrete traction data as a function of separation (Methods section) and a triangular form is not prescribed in our ML model.

More interestingly, we investigate our model performance on a different form of T–S relation, namely 3–9 Leonard-Jones (L-J) potential^20^, which is not included in the training process and the L-J potential form is not given during ML prediction. For conventional fittings or inverse properties extractions, predefined forms for the target are generally required. This is a constraint that one needs to make assumptions or use trial and error to determine a proper function form for fitting. In contrast, our ML model can be used directly for prediction instead of predetermining the form for the targeted T–S relation. The ML model is trained based on purely triangular T–S relations, in which the L-J potential form is not given during the training process. Three examples of F–D curves generated via different L-J potentials are shown in Fig. 2c and their corresponding predicted results are in Fig. 2d. Although variations are observed at the softening process around separation of 100–250 μm, the predictions capture key features of L-J potential, including the gradual transition near maximum traction and long tails of the T–S relation (Fig. 2d). This indicates the advantage of data-driven ML that the data features can be captured without making assumptions of targeted fitting forms, as well as a capability to predict different forms of T–S relations out of the training data domain.

To generalize our ML model to different materials and dimensions of DCBs, we introduce the normalized force and displacement that ${\boldsymbol{\bar{F}}}=\boldsymbol{F}{\boldsymbol{a}}^{2}/(\boldsymbol{E}{\boldsymbol{h}}^{3}\boldsymbol{t})$ and ${\boldsymbol{\bar{d}}}=\boldsymbol{d}/\boldsymbol{a}$, where *E* is the Young’s modulus of the beams, *a* is the initial crack length, *h* is beam thickness, and *t* is beam width. Since the FEA database for ML model training is established based on the linear elastic deformation assumption and the maximum normalized force of 0.001 < ${\stackrel{-}{\boldsymbol{F}}}_{\boldsymbol{m}\boldsymbol{a}\boldsymbol{x}}$<0.04, our ML model would be applicable to predict the T–S relations within these limitations.

### Experimental validation

**Figure 3:**
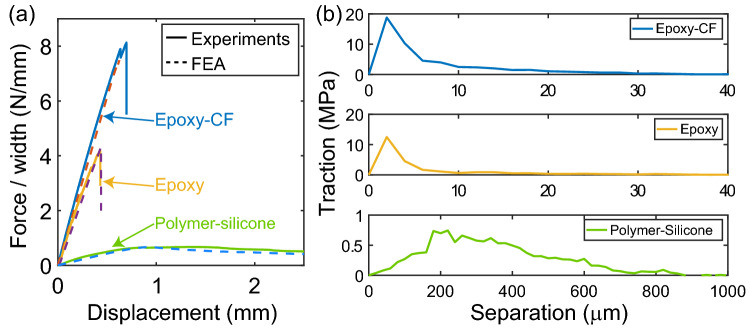
DCB experimental data for ML model validation. (**a**) Experimental F–D curves and FEA results using (**b**) T–S relations trained from experimental F–D curves for epoxy, epoxy-CF, and polymer-silicone interfaces.

To validate the T–S relation established from the XGBoost ML approach, we perform DCB experiments for three material systems, namely epoxy, epoxy-CF composite, and polymer-silicone interfaces. These experiments yield the F–D curves plotted in solid lines in Fig. 3a. The F–D curve of polymer-silicone interface has a compliant stiffness and a gradual softening process after maximum force at around 1 mm, indicating a gradual debonding at interface along the crack. Both pure epoxy and epoxy-CF composite have stiff and brittle interface. The drops in F–D curves indicate catastrophic failures of interfaces in both materials. These three material systems are selected to validate the application of our ML model on both hard and soft interfaces, as well as single component and composite materials.

Harnessing our trained ML model, T–S relation for each material system is generated individually based on the experimental F–D curves (Fig. 3b). Stiff and short-range interface interaction is seen for epoxy and epoxy-CF, with strength of the former (12.5 MPa) is 66% of the later (18.8 MPa). Polymer-silicone interface shows a compliant and long-range T–S relation, which is expected because the silicone can be largely deformed before breaking. Subsequently, the T–S relations are used in FEA simulations to obtain F–D curves (dashed lines in Fig. 3a). We observe that the F–D curves from simulations are in agreement with experimental F–D curves in all three cases. The comparisons demonstrate that the accurate T–S relations are predicted from experimental data using our ML model.

### Models performance

**Figure 4:**
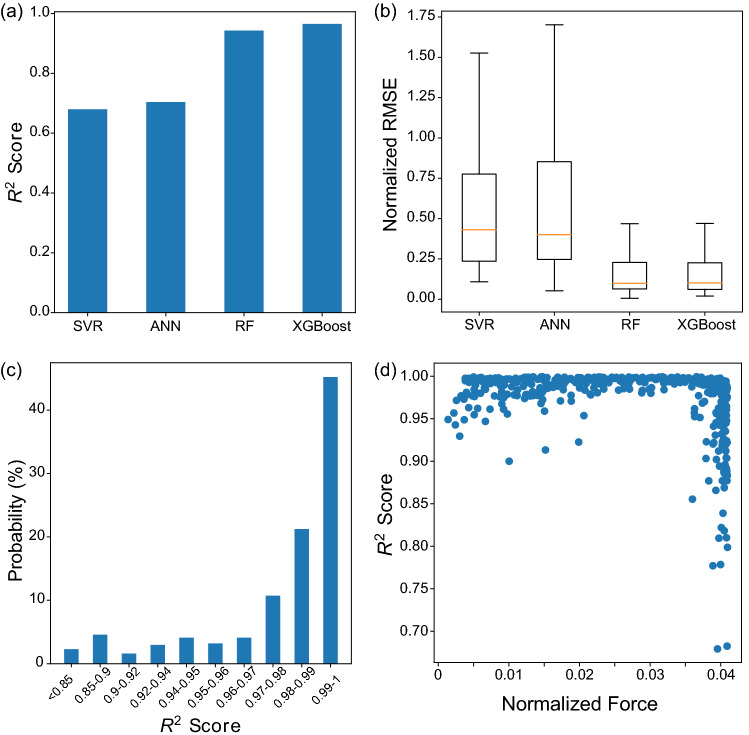
Performance of different machine learning methods. (**a**) *R*^2^ score and (**b**) normalized RMSE for four different methods. XGBoost shows the highest *R*^2^ score and lowest RMSE. (**c**) Distribution of *R*^2^ score for the XGBoost model and (**d**) relation between *R*^2^ and maximum normalized forces.

We investigate other ML models that are support vector regression (SVR), artificial neural network (ANN), and random forest (RF) to compare with the XGBoost model. The performance of models is evaluated on the basis of coefficient of determination (*R*^2^ score) and root mean square error (RMSE), which is normalized by maximum interfacial traction in each test case. As illustrated in Fig. 4a and b, XGBoost outperforms other models with the highest *R*^2^ score (*R*^2^ = 0.988) and lowest normalized RMSE. Though comparable performance is seen in RF model, the XGBoost is the most efficient and takes only half of training time than others.

To further evaluate the performance in XGBoost model, we show the distribution of *R*^2^ scores for all testing samples in Fig. 4c. *R*^2^ scores are concentrated at high values (near to 1) and more than 40% predicted samples lie in the 0.99–1 range. More than 84% samples have a *R*^2^ score more than 0.95. To identify the low performance cases, we exhibit individual *R*^2^ score for each test case as a function of maximum normalized force in Fig. 4d. The normalized force is $\stackrel{-}{F}\, =\, F{a}^{2}/(E{h}^{3}t)$, where *a* = 25 mm is the distance of loading location to initial crack tip, *E* = 2 GPa is the Young’s modulus, *h* = 5 mm is the thickness, and *t* = 1 mm is the width in our DCB models for simulations. When the test data has a maximum force approaching the limits of force range in our training database (0.001 < ${\stackrel{-}{F}}_{max}$<0.04), the *R*^2^ score decreases significantly. For lower bound, negative *R*^2^ scores are observed (not shown in the figure), indicating the prediction is even worse than a simple average of data. For upper bound, *R*^2^ scores distribute from 1 to less than 0.7. Between these two bounds, the ML predictions have *R*^2^ between 0.95 and 1 with only a few exceptional cases.

Based on our trained XGBoost model, we develop a code package that allows users to predict T–S relations by simply inputting F–D curves from DCB experiments or simulations. This package, including trained model file, python source codes, example data, and user guideline, is available to be downloaded^37^. This program can establish T–S relations without setting up a new database or training ML models again.

### Predictions of interface imperfections

**Figure 5:**
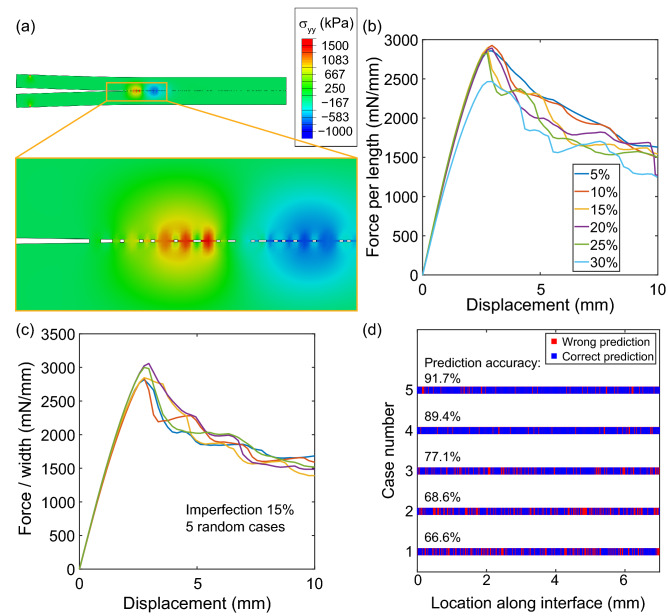
Prediction of interfacial imperfection by machine learning. (**a**) An example of stress distribution at interface with imperfections in a FEA DCB test. Force–displacement curves obtained from cases with (**b**) different imperfection ratio and (**c**) different locations of imperfections. (**d**) Examples of imperfection locations predictions using XGBoost classifier. The average prediction accuracy is 80.9% over 312 testing cases.

The imperfections can affect the stress distribution at the interface remarkably and thus leads to different crack propagation and failure behaviors^38,39^. In addition to characterizing T–S relation at perfect interfaces, we harness the FEA-ML method on identifying interface imperfections. As one example shown in Fig. 5a, interfacial voids are generated in our FEA models as imperfections. Local stress concentration depends on imperfection distribution, so does the macroscale F–D curves. Both the ratio and the locations of these imperfections result in different maximum forces, loading slopes, and force fluctuations in F–D curves (Fig. 5b, c). Such force fluctuation may simply be smoothed or considered as noise, especially in experiments, without relating such behavior to interface imperfections, not mention to precisely identify imperfection locations.

To identify the locations of interfacial imperfections, the interface (70 mm in length) is discretized into 0.2 mm intervals and analyzed as target features. Then, we use four different classification methods to establish ML models trained on F–D curves and imperfection locations correlated data. The four methods, including support vector classification (SVC), ANN classification, RF classification, and XGBoost classification, result in close prediction accuracy between 81–82% (see Methods section for details). The XGBoost classification model results in an average accuracy of 80.9 ± 8.6% predicting bonded and imperfect locations along the interface for 312 random cases. Five cases with prediction accuracy from 67 to 92% are shown as examples in Fig. 5d, indicating correct and wrong predictions along interface and their locations.

## Conclusions

In conclusion, we have demonstrated the process of characterizing interface adhesive properties and imperfections using a combination of FEA and ML. We use advanced ML models framing with multi-output regressors to train on a FEA database, simulating DCB tests with interfacial stiffness and strength over 5 and 6 orders of magnitude, respectively. Our optimized XGBoost model predicts T–S relations with a coefficient of determination of 0.988. We use 3D printed DCB specimens of epoxy, epoxy-CF, and silicone-polymer interfaces to experimentally measure F–D curves, extract T–S relations by our ML model, and then validated by the F–D curves that are consistent in both experiments and FEA. Furthermore, we leverage this FEA-ML approach to illustrate the capability of detecting imperfection locations along interface with an accuracy of 80.8 ± 8.6%. Finally, we develop a Python code package including our trained ML model for establishing T–S relations for other material systems.

## Methods

### Database collection via FEA

Double cantilever beam (DCB) specimens were established as 2D plane strain models in Abaqus® 2020. A layer of cohesive elements (70 mm × 0.1 mm, element type: COH2D4) was sandwiched between two solid beams (100 mm × 5 mm, element type: CPE4R). Mesh convergence check was done by carrying out simulations with 4 meshing sizes of about 1.5 times differences in sequence, namely 0.23, 0.15, 0.1, and 0.07 mm. To mimic the DCB tests, a vertical displacement of 10 mm was applied on one beam at a distance of 25 mm away from the initial crack tip and a fixed boundary condition was applied at the corresponding location on another beam. Force was collected as a function of displacement from the simulations. To establish the database, triangular traction–separation relation was used with normal stiffness ranging from 20 kPa/mm to 2 GPa/mm, maximum strength from 0.2 kPa to 200 MPa, and energy dissipation from 2 mJ/m^2^ to 60 kJ/m^2^. The upper and lower bounds of these parameters were extensively selected to allow the database to include large enough ranges for ML. In total, 1500 simulations were performed, and their corresponding T–S relations and F–D curves were discretized into 500 and 1000 features, respectively, for the purpose of ML.

For non-triangular traction–separation relations, including Lennard–Jones potential and ML results from experiments, tabular function was utilized when defining the damage evolution for cohesive elements. For interfaces with imperfections, a Matlab® code was generated to read Abaqus input files, randomly select and remove interfacial cohesive zone elements, and rewrite new input files by batch. Additional 780 cases with interfacial imperfection ratio 5% to 30% were produced. For each case, a F–D curve and the locations of imperfections were generated for ML.

### Predictive ML models

Common supervised ML regression models include SVR, RF, ANN, and boosted tree based models, such as XGBoost^40^. In this work, we particularly concentrated on the XGBoost algorithm as it showed competitive performance compared to other advanced ML algorithms^36^. Additionally, XGBoost algorithm delivers a good generalization and speed through building tree-based ensemble technique^35^. We established our multi-output regression models according to XGBoost^35^ and scikit-learn^41^ ML packages. The XGBoost model was an association of gradient decision trees with enhanced speed and performance^35^. It created a model where residuals were calculated from the prior model and combined to forecast the final prediction. We split the datasets by 70/30 rule, where we trained the ML model using 70% data. The remaining 30% was the testing data to evaluate each model. Before training the models, we performed an extensive grid search using training data to determine optimal parameters. In grid search, we set maximum depth in a range between 6 and 18, learning rate 0.06–0.4, and subsample 0.2–0.6 with fourfold-cross validation in order to obtain reliable results. We determined the optimal parameters for XGBoost as linear regression for the objective function, 16 for the maximum depth of the decision trees, 0 for γ, 0.5 for subsample to control overfitting, and 0.08 for the learning rate. We calculated median *R*^2^ score for training data along with the test data. The median of *R*^2^ score for the training and test data were 0.999 and 0.988, respectively. The difference between training and test data was about 1% and *R*^2^ score on test was close to 1, which indicates the model was neither overfitting nor underfitting^42,43^. ML models were applied to 1500 samples, where each sample contained 1000 input features of F–D curves and 500 output features of T–S relation. To deal with multiple features, we tested both direct and chained multi-output regression methods. The detailed algorithm flows can be found in literatures^44,45^. In direct multi-output regression, each feature in the output was treated as an independent target variable for prediction. Each sample was divided into 500 (number of output features) separate problems. In chained multi-output regression, it creates a linear sequence of models for each sample, where predicted output features in previous steps were used together with input features to predict next feature^41,46–48^. Therefore, our multi-output models with 1500 samples and 500 output features each are equivalent to a data size of 1500 × 500 = 750,000 in single output models. We have applied both approaches and the direct multioutput regressor shows slightly better performance. Convergence tests were carried out on XGBoost model, using 10 sample sizes from 105 to 1050 samples and resulting in a converged *R*^2^ score at 0.988.

In addition to XGBoost, we employed other three algorithms for comparison, namely SVR, ANN, and RF^49–51^. Optimal parameters for all these models were selected via grid search. In SVR, we used radial basis function (rbf) kernel with *C* = 1000 as regularization parameter and ɛ = 0.001 that associated with a training loss function. In RF algorithm, multiple decision trees were constructed to calculate the mean from all the trees. We found that 100 decision trees and maximum depth of 10 for those trees were optimal from a grid search. Finally, we used ANN that had capabilities of learning from sequential data and consisted of several hidden layers^52^. In this work, we applied 2 hidden layers with 200 neurons in each.

Regarding the interface imperfection task, we used XGBoost classifier for predicting locations of imperfection, where the label 1 indicated as perfectly bonded interface and 0 indicated as an imperfection (void). Similar to the regression models, we used 780 samples and 350 output features each to establish our multi-output classification models. The convergence tests of 7 sample sizes from 78 to 546 samples were performed and accuracy converged to 0.808. To work with the classification task, we set logistic regression as the objective function and applied another grid search. The grid search set a range of 200–350 for the number of trees, 6–10 for the maximum depth, 0.005–0.1 for the learning rate, and 0.5–7 for L2 regularization, respectively. As a result, we used linear regression with 300 for optimal number of trees, 6 for maximum depth, 0.008 for the learning rate, and 5 for L2 regularization. In addition, three other ML models, specifically SVC, ANN classification, and RF classification, were tested. We utilized a grid search mechanism as before to obtain the optimal parameters. For RF classifier, we used 100 as number of trees in the forest, 6 for maximum depth of trees, 5 for minimum number of samples required to split an internal node, 2 for minimum number of samples to be required at a leaf node. In ANN classification, we applied one hidden layer with 100 neurons, ‘tanh’ activation function for the hidden layer, *α* = 0.0001 for L2 regularization, and a stochastic gradient descent (sgd) solver for weight optimization. For SVC model, we used the rbf kernel, γ = 1 for kernel coefficient, and *C* = 0.1 for regularization parameter^41,49–51^.

### Double cantilever beam (DCB) experiments

DCB specimens of different material systems were prepared by 3D printing. The material systems included epoxy-epoxy, epoxy-carbon fiber (CF) composites, and polymer-silicone interfaces. We 3D printed epoxy and its composites into DCB specimens using direct ink writing (DIW) method. The inks were prepared based on our previously reported literature^53^. Briefly, epoxide resin (Epon 826®) was mixed with cross-linkers (Epikure W®, 25 wt%, and Epikure 3140®, 5 wt%), and silica nanoparticles (EH-5®, 13 wt%) using a planetary mixer. Carbon fibers (10 wt%) were added to the mixture to prepare the epoxy-CF composite ink. The as-prepared inks were 3D printed by DIW technique using a Hyrel 3D SR Engine® printer with a nozzle size of 0.3 mm, layer height of 0.15 mm, and a printing speed of 10 mm/s. After printing, the materials were thermally cured at 150 °C for 10 h. For polymer-silicone samples, we used stereolithography (SLA) to 3D print polymer beams (Formlab® Tough resin), which were then washed by isopropyl alcohol (IPA) and cured at 60 °C for 60 min. Then, a layer of silicone glue was applied at interface and cured at room temperature for at least 2 h. The polymer-silicone samples had a beam thickness of 5 mm, a beam width of 20 mm, a length of 100 mm and an initial crack length of 25 mm (ASTM D5528 standard). A scaling factor of 0.8 was used for epoxy and epoxy-CF samples to reduce the 3D printing time. Different material properties were considered in FEA validations, where *E*_*pol*_ = 1.5 GPa for SLA printed polymer beams, E_epoxy_ = 1.6 GPa and E_epoxy-CF_ = 2.2 GPa for DIW printed epoxy and epoxy-CF samples, respectively. DCB tests were performed via a universal tensile machine under displacement control (0.1 mm/s) up to 10 mm. F–D curves are collected at 10 pts/s.
